# Heterochromatin organization and phase separation

**DOI:** 10.1080/19491034.2022.2159142

**Published:** 2023-01-29

**Authors:** Hui Zhang, Weihua Qin, Hector Romero, Heinrich Leonhardt, M. Cristina Cardoso

**Affiliations:** a Cell Biology and Epigenetics, Department of Biology, Technical University of Darmstadt, Darmstadt, Germany; b Human Biology and Bioimaging, Faculty of Biology, Ludwig Maximilians University Munich, Planegg-Martinsried, Germany

**Keywords:** DNA methylation, heterochromatin, histone H1, histone modifications, HP1, liquid-liquid phase separation, MeCP2, RNA binding proteins, X chromosome inactivation

## Abstract

The eukaryotic nucleus displays a variety of membraneless compartments with distinct biomolecular composition and specific cellular activities. Emerging evidence indicates that protein-based liquid–liquid phase separation (LLPS) plays an essential role in the formation and dynamic regulation of heterochromatin compartmentalization. This feature is especially conspicuous at the pericentric heterochromatin domains. In this review, we will describe our understanding of heterochromatin organization and LLPS. In addition, we will highlight the increasing importance of multivalent weak homo- and heteromolecular interactions in LLPS-mediated heterochromatin compartmentalization in the complex environment inside living cells.

## Chromatin organization

The eukaryotic nuclei are heterogeneous containing multiple membraneless organelles with distinct compositions, metabolism, and dynamics [1]. In eukaryotic cells, chromatin is hierarchically organized into distinct domains with different epigenetic modifications, gene expression profiles, and chromatin dynamics [2].

**Figure 1.**
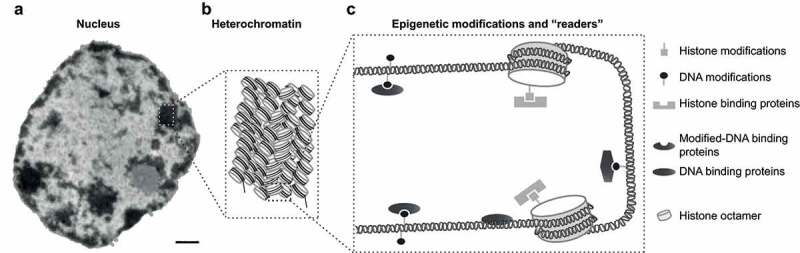
Heterochromatin recognition and epigenetic modifications.

Based on the compaction levels, chromatin can be classified into euchromatin and heterochromatin. *In vivo*, the two components can be distinguished by differential DNA staining under the microscope (Figure 1a). Euchromatin is composed of mainly transcriptional active genes and hallmarked with low DNA methylation and high histone acetylation (H3K4ac) [3,4]. Heterochromatin is the highly compacted form of chromatin with restricted accessibility of DNA and includes mainly inactive genes and repeat elements (Figure 1b). Heterochromatin is hallmarked by high DNA methylation and trimethylation at histone 3 lysines 9 and 27 (H3K9me3 and H3K27me3) (Figure 1c) [3,5–7].

(a) Transmission electron microscopy image of a mouse liver cell nucleus with electron-dense regions corresponding to heterochromatin. Scale bar = 0.5 µm. (b) Graphical representation of densely packed nucleosomes within heterochromatin. (c) Simplified cartoon showing epigenetic modifications and corresponding ‘readers’ within heterochromatin regions. NU: nucleolus; EC: euchromatin; HC: heterochromatin. Symbols are as indicated.

In mouse and *Drosophila* cells, the constitutive heterochromatin around the centromeres from multiple chromosomes clusters together forming pericentric heterochromatin compartments (also named chromocenters) during the interphase. In the last decades, several groups have investigated the composition, function, and dynamics of heterochromatin compartments [8–18].

Heterochromatin plays an essential role in nuclear architecture, genome stability, expression, and organization [8,11,19–21]. Dysregulation of the heterochromatin compartments is associated with senescence and aging, which are hallmarked by deregulated lamin B1 (LMNB1) [22]. Low LMNB1 leads to the loss of H3K9me3 at the nuclear periphery and contributes to the reactivation of inflammatory genes [23]. Besides, disease-causing lamin A (LMNA) mutations lead to globally reduced levels of LMNB1, HP1α, H3K9me3, and H3K27me3 [24–26].

Nevertheless, the underlying mechanisms regulating heterochromatin formation, maintenance, and/or dynamics are still far from clear. Recent studies indicate a role of liquid–liquid phase separation (LLPS) in heterochromatin compartmentalization.

## Liquid-liquid phase separation and human diseases

**Figure 2.**
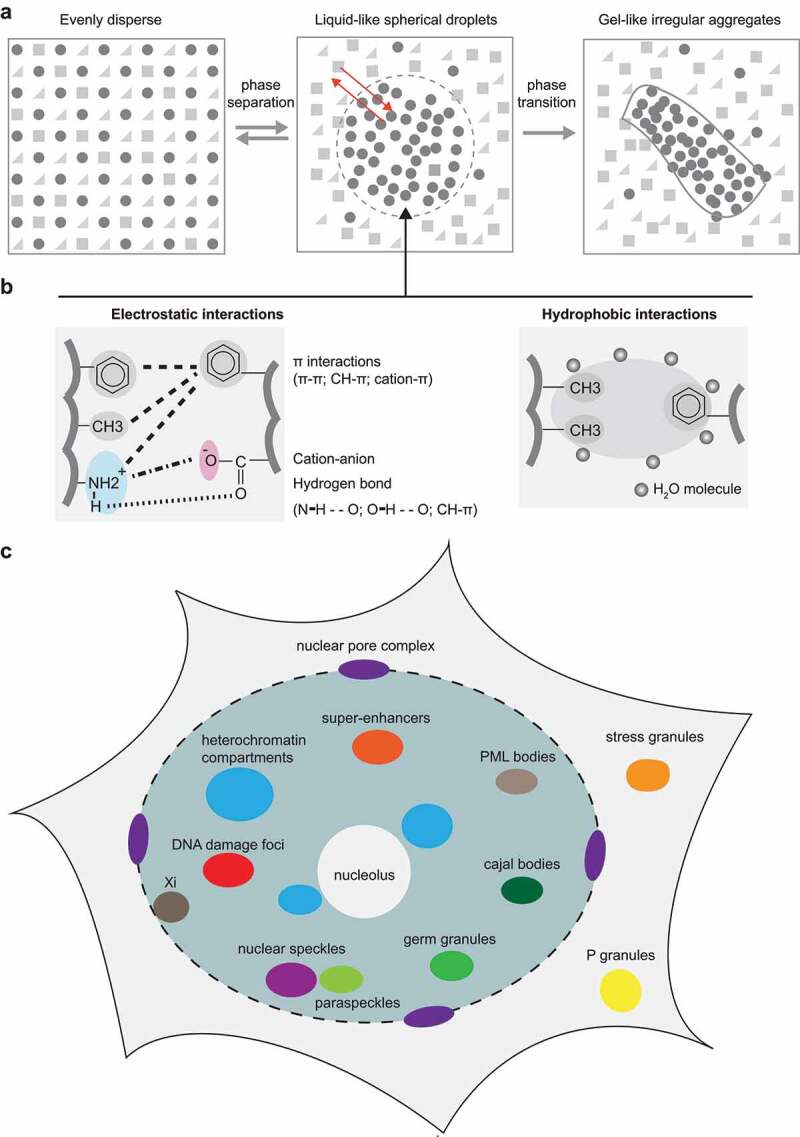
Multivalent interactions-directed liquid-liquid phase separation in vitro and in vivo.

Distinct from membrane-bound organelles in cells, which are surrounded by a phospholipid membrane, membraneless organelles lack a definite boundary and are, thus, very dynamic having the ability to condense and/or dissolve upon changing conditions. This phenomenon is named liquid–liquid phase separation (LLPS). The protein-based LLPS forms liquid-like spherical droplets (condensates, compartments, or foci), which are characterized by high protein concentration (dense phase), reversibility, and molecular exchangeability with the surrounding milieu (dilute phase) (Figure 2a).

(a) In a mixture containing different molecules, originally evenly dispersed unstructured protein(s) demix to form two phases, the dense phase, and the dilute phase. This process is called liquid–liquid phase separation (LLPS). The dense phase (droplets) shows the properties of reversibility, spherical morphology, and dynamic molecule exchange between the two phases. Under certain conditions (such as proteins with specific disease-related mutations), the liquid-like droplets further develop into irregular aggregates with slow/no molecule dynamics, a state which is irreversible. This process is called phase transition. (b) LLPS is mainly driven by multivalent interactions including electrostatic interactions and hydrophobic interactions. The electrostatic interactions include cation–anion interaction, π interactions, and hydrogen bond. The hydrophobic interactions happening between hydrophobic amino acids with methyl or aromatic groups can also contribute to the formation of condensates by excluding water molecules and, thus, being brought together. (c) LLPS is now characterized as a common mechanism underlying the formation and dynamic regulation of a variety of membraneless organelles within the cytoplasm and nucleus. Xi: inactive X chromosome.

LLPS was originally applied in membrane science to describe and characterize the demixing process during asymmetric membrane formation in polymer solution [27]. *In vitro*, the lysozyme protein was also shown to undergo demixing and form liquid droplets at high lysozyme concentrations, which promoted its crystallization process (a process called phase transition) [28]. Yet, the involvement of LLPS in cellular activities was not described until 2009, when Brangwynne et al. proposed that germline P granules are liquid-like spherical droplets with fast exchange dynamics, fusion, and fission properties [29]. This indicates that P granules are probably regulated by LLPS and further that LLPS might underlie the formation and regulation of multiple membraneless organelles with different cellular activities.

Proteins that could undergo LLPS often contain intrinsically disordered regions (IDRs) [30]. Distinct from structured proteins or protein regions the IDRs are characterized by biased amino acid composition (more hydrophilic and less hydrophobic residues [31]), lack a fixed and ordered structure in the absence of interactive partners (nucleic acids, proteins, etc.), and adopt flexible conformations based on binding partners. IDRs are also involved in weak multivalent interactions [32–34].

The LLPS is driven by multivalent weak and transient interactions among IDRs including electrostatic interactions (charge–charge, charge-π, CH-π, π-π, and hydrogen bonds) and hydrophobic interactions (happening in nonpolar amino acid residues with hydrophobic side chains, such as methyl or aromatic groups) (Figure 2b) [35–38].

Further, the droplets formed by LLPS might undergo a further transition to less reversible gel-like and, at extreme conditions, solid condensates. This process is named phase transition (Figure 2a, right). The driving force underlying phase transition is not clear, but likely sequence coded. Phase transition plays a role in cellular activity. For example, in yeast, poly(A)-binding protein (Pab1) undergoes LLPS under heat shock and forms grainy, amorphous particles with little or no dynamics at higher temperatures and lower pH [39]. This suggests that the gel-like condensates formed by Pab1 are possibly an adaptive response during stress.

The abnormal phase transition is tightly correlated with human diseases in most conditions studied. Parkinson’s disease is such an example and is hallmarked by α-synuclein (α-Syn) aggregation and amyloid plaque formation [40]. Both *in vivo* and *in vitro* results show that α-Syn can form liquid-like droplets, which eventually transform into amyloid-hydrogel-containing oligomers and fibrillar species [41]. In particular, familial Parkinson’s disease mutations promote the LLPS and phase transition to aggregated states [41]. Similar phenomena are also observed for tau, another component involved in Alzheimer’s disease [42].

The protein-based LLPS is now identified as a general mechanism underlying the organization of various membraneless organelles in the cytoplasm (P granules, stress granules, etc.) and nucleus (super-enhancers, heterochromatin compartments, etc.) with well-delineated physicochemical boundaries but without phospholipid membrane barriers (Figure 2c).

## LLPS in (hetero)chromatin compartmentalization

In recent years, an increasing amount of chromatin-binding proteins were shown to have the ability of LLPS in both euchromatin and heterochromatin regions, while the chromatin itself exhibits solid-like state *in vivo* due to its large size [43]. On the other hand, the ‘jelly-like’ chromatin also provides a scaffold for the LLPS of multiple chromatin-binding proteins [43], which will be discussed.

*In vitro*, nucleosome arrays (NAs) were shown to undergo histone tail-dependent liquid-like phase separation in physiologic salt conditions [44]. This could be promoted by the linker histone H1, controlled by linker DNA length, and disrupted by histone acetylation. Furthermore, NAs with acetylated histones were reported to form a new liquid phase with multi-bromodomain proteins recognizing the acetylated histones and these condensates exhibited distinct properties compared to droplets formed by unmodified histones. This indicates a role of LLPS in the segregation of euchromatin and heterochromatin [45,46].

In euchromatin, LLPS plays a role in the local enrichment of certain factors that are essential for certain nuclear activities, including DNA replication, transcription, damage repair, and alternative splicing (Figure 2c) [47–55]. For example, the super-enhancer-associated transcription coactivators BRD4 and MED1 were shown to form liquid-like condensates *in vivo* and *in vitro* in an IDR-dependent manner [48]. The condensates could then recruit multiple transcriptional factors (such as OCT4, SOX2, and NANOG) and the transcriptional machinery [47,48]. Besides, disrupting the phase separation properties of OCT4 with MED1 via mutation of acidic residues (mutating negatively charged aspartic/glutamic acids to nonpolar alanine) also decreased the ability of OCT4 to activate gene expression [47].

**Figure 3.**
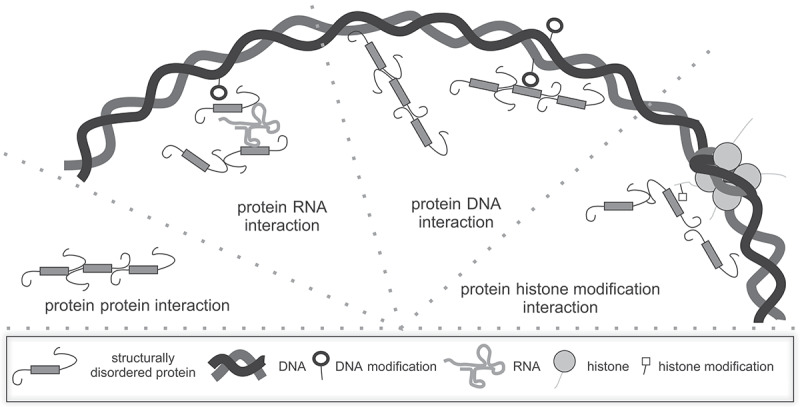
Possible interactions occurring in heterochromatin compartmentalization by liquid-liquid phase separation.

LLPS was shown to drive heterochromatin compartmentalization, such as 1) pericentric heterochromatin foci which could be mediated by heterochromatin protein 1 (HP1) [13], 53BP1 [56], methyl-CpG binding protein 2 (MeCP2) [57–59], and scaffold attachment factor B (SAFB) [60]; 2) X chromosome inactivation which is initiated and maintained by RNA-binding proteins [61]; 3) telomeres whose multicomponent liquid-like state is regulated by TRF1/2 driven LLPS [62]. Moreover, LLPS could be regulated by distinct interactions, including homo/heterotypic interactions between proteins [56,63,64], protein-DNA [57–59], and protein-RNA [60,62] (Figure 3). In the following, we discuss and illustrate these different modes of heterochromatin compartmentalization (see summary in Table 1).

Either homo or hetero protein–protein interactions are a prerequisite for liquid–liquid phase separation processes. Additionally, the proteins involved can interact with other factors, including: i) DNA, either naked DNA or modified DNA such as 5mC; ii) RNA; and iii) histone modifications such as H3K9me3. These interactions enhance the LLPS formation and/or allow the formation of the LLPS compartments in the heterochromatic regions.

**Table 1.** Summary of LLPS studies regarding heterochromatin compartmentalization.

		In vitro	In vivo	Ref.
	Factor	conditions/mutants/PTMs/ interactors	compartment & protein dynamics (properties)	
RNA- dependent	Xist (E-repeat)	no LLPS by itself E-repeat of Xist recruits PTBP1 and promotes protein LLPS via protein-protein and protein-RNA interactions	Xist foci tethers to chromosomal locations with high affinity and limited movements E-element depletion (Xist∆E) increases Xi foci and causes inheritable gene silencing	[61,98]
	PTBP1	> physiological concentration Xist E-repeat of Xist: promotes CELF1: promotes	wt PTBP1 rescues the phenotypes in Xist∆E cells, while PTBP1 mutants could not FRAP partial recovery	[61,98]
	TDP-43	salt and protein concentration dependent RNA (promotes) TDP-43 mutations (attenuate)	wt TDP-43 rescues the phenotypes in Xist∆E cells, while TDP-43 mutations could not	[61,99,100]
DNA- dependent	MeCP2	salt: attenuates protein concentration dependent protein regions: basic (required), C-t (not required) isoform dependent (E2>E1) mutations: attenuate crowders: promote DNA: promotes 5mC-DNA: dose-dependent NA: promotes 5mC-DNA-NA: dose-dependent FRAP partial recovery single-molecule tracking microscopy: crowders (constrains), DNA (does not affect), 5mC-DNA (immobilizes)	FRAP partial recovery increase individual size (also limited by 5mC-DNA levels) and reduce compartment number single-molecule tracking microscopy: concentration (constrains), mutations (increase), 5mC (constrains) mutations: affect recruitment, compartment size and increase MaSat RNA transcription H1 is excluded from MeCP2 compartments	[57–59,74]
Histone- dependent	Histone H1	DNA: promotes NA: promotes		[44],[88,91],[101]
	HP1α	salt: attenuate phosphorylation: promotes hinge swap mutants: attenuate Sgo1: promotes LBR: attenuate SUV39H1/KAP1: promotes DNA: promotes NA: promotes	FRAP partial recovery	[59],[13,16,50,84,85,102]
	HP1β	salt: attenuate phosphorylation: promotes hinge swap mutants: attenuate/promote core histones: promote SUV39H1/KAP1 together with nucleosome extracts: promote	FRAP partial recovery mutations: affect recruitment & size	[13,16,50,84,85,102],[63,64]
	HP1γ	hinge swap mutants: attenuate/promotes KAP1/TRIM28 together with chromatin extracts: promote DNA: promotes		[13,16,50,84,85,102]

Abbreviations: 5mC - 5-methyl-cytosine; C-t - C-terminus; FRAP - fluorescence recovery after photobleaching; MaSat - major satellite; NA - nucleosomal array; wt - wild type.

## Protein-RNA-dependent LLPS and function in X chromosome inactivation

**Figure 4.**
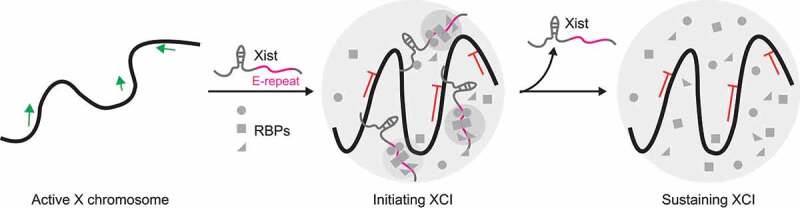
Model of inactive X chromosome compartment formation via Xist-initiated protein condensation and Xist-independent inactive X chromosome maintenance.

LLPS is involved in *Xist* long non-coding RNA initiated X chromosome inactivation (XCI) by recruiting ubiquitous *Xist*-RNA binding proteins (RBPs) via the multivalent E-repeat element of the *Xist*. Yet, the maintenance of the inactive X compartment is independent of *Xist* RNA (Figure 4) [61].

Initially, the Xist RNA recruits RNA-binding proteins (RBPs) across the X chromosome via the multivalent E-repeat motif and induces the condensation and inactivation of the X chromosome via homo- and heterotypic protein–protein interactions. This process is called X chromosome inactivation (XCI). After this, the condensates can be sustained in the absence of Xist.

XCI is the transcription repression of one X chromosome in female cells to ensure equivalent expression levels of X-located alleles between male and female cells [65]. Heterochromatic inactive X chromosome (Xi) is then condensed into a compacted structure, which is often located close to the nuclear periphery or nucleolus with a complex but unique composition [66,67]. XCI is divided into three distinct stages: initiation, establishment, and maintenance together with a transition from *Xist*-dependent silencing to *Xist*-independent during embryonic stem (ES) cell differentiation [68,69].

The long non-coding RNA *Xist* contains multiple functional elements featured with repetitive motifs including the E-repeat element [68]. The E-repeat element is a C, U, and G-rich RNA sequence and functions as a multivalent binding platform for PTPB1, MATR3, TDP-43, and CELF1 [61,69–72].

In ES cells, disrupting *Xist*-protein interactions via the *Xist*∆E (E-repeat depletion form of *Xist*) or depletion of PTBP1, MATR3, TDP-43, and CELF1 resulted in more dispersed *Xist* foci with increased foci number. This could be rescued by targeted expression of the above mentioned genes [61]. This led to the proposal that the E-repeat-mediated protein recruitment promotes the integration of multiple *Xist* transcripts into individual *Xist* foci, their localization and stabilization. During differentiation, the X-chromosome gene silencing state could not be maintained in cells expressing *Xist*∆E, accompanied by decreased DNA and H3K27me3 intensities within Xi [61], indicating that the E-repeat is essential for heritable gene silencing and Xi compaction. Moreover, the E-repeat-binding proteins exhibited LLPS properties, including PTBP1 [38], TDP-43 [71], and MATR3 [73]. *In vitro*, the PTBP1 LLPS was enhanced by E-repeat RNA and CELF1, whereas the TDP-43 or MATR3 phase separations were diminished by point mutations [61]. *In vivo*, the point mutations that abolished the LLPS of TDP-43 or MATR3 failed to rescue the gene silencing in the differentiating cells with *Xist*∆E expression [61]. Thus, the E-repeat-mediated LLPS of PTPB1, MATR3, TDP-43, and CELF1 via homo/hetero-interactions compartmentalize *Xist* and enforce X-chromosome gene silencing. Moreover, CELF1 enrichment at Xi remained upon depletion of *Xist* [61], indicating that protein condensates containing PTPB1, MATR3, TDP-43, and CELF1 persist in a *Xist*-independent manner in the *Xist*-independent stage.

## Protein-DNA-dependent LLPS and function in heterochromatin

**Figure 5.**
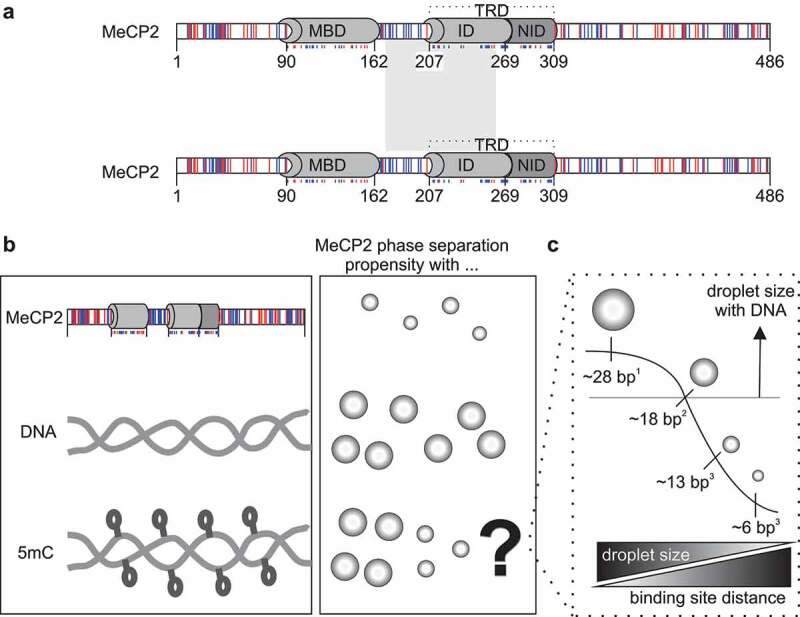
MeCP2 mediated liquid-liquid phase separation.

Methyl-CpG binding protein 2 (MeCP2) is the most studied member of the methyl-CpG binding domain (MBD) containing proteins, which recognize the 5-methyl-cytosine (5mC) modification in the DNA. MeCP2 has been shown to form LLPS in the absence of DNA in physiological concentrations [58,59]. MeCP2-driven LLPS requires self-interactions and is mainly based on electrostatic forces [58]in particular, the ones derived from basic regions [59] located between the MBD and the NID domains [45] (Figure 5a). DNA was shown to promote MeCP2 LLPS formation in a concentration-dependent manner (Figure 5b), while the nucleosomes were shown to have a negative effect compared to the same size naked DNA [57–59].

*(a) MeCP2 LLPS is driven predominantly by electrostatic self-interactions mainly in the region from the end of the methyl-CpG binding domain (MBD) to the N-CoR interacting domain (NID). Color coding: blue for basic amino acids and red for acidic amino acids. TRD: transcriptional repression domain; ID: intervening domain. (b) Overview of MeCP2 phase separation propensity in the absence of DNA or in the presence of unmodified or methylated DNA. (c) In view of the differences found between publications, we compared the distance between MeCP2 binding sites and the results observed in each publication. ^1^* [59] *^2^* [57,74] *^3^* [58].

There is a certain controversy concerning the results obtained regarding the effect of 5mC containing DNA in MeCP2 LLPS. In all studies, 5mC containing DNA promoted LLPS formation, but they showed different outcomes when comparing the effect of 5mC on the size of the droplets relative to the respective unmethylated DNA. A potential explanation for this phenomenon could be the different distances between MeCP2 binding sites (i.e., 5mC density) in the different studies (Figure 5c). With a distance of 28 bp [59], MeCP2 droplet size was increased compared to the corresponding naked DNA in all conditions studied, while when this distance was reduced to 18 bp [57,74], there were some conditions in which the size was unaffected or even restricted. Moreover, with a distance further reduced to 13 or even 6 bp [58], most of the situations led to restriction of droplet size with greater effect with shorter binding site distances. For comparison, in mouse cells, major satellite repeats at pericentric heterochromatin have MeCP2 binding sites spaced from 5 to 14 bp [75].

Recent modeling studies suggest that immobilization of a protein capable of LLPS reduces the compartment size [76], and this could explain what happens to MeCP2 LLPS by introducing 5mC in the DNA. In addition, quantification of 5mC in the heterochromatin suggested that around 65–85% of the CpGs located in heterochromatin are methylated in proliferating mouse cells [75,77] and likely more in differentiated cells, which could be a mechanism of the cells to prevent that all heterochromatin fuse into one unique subnuclear compartment. This phenomenon has been reported in Purkinje cells during the development of mouse cerebellum. Together with a global DNA demethylation process between postnatal day 7 (P7) and P29 [78], there was an increase of the heterochromatin compartments size in contrast with what happened in the granular cells that surround the Purkinje cells, where there is an increase in 5mC levels. Indeed, MeCP2 mobility analysis showed a decreased bound fraction in Purkinje cells compared to the granular cells [79].

In addition to limiting the compartment size, 5mC also plays a role in targeting MeCP2 to heterochromatin regions. MeCP2 mutants that affect the MBD binding to 5mC are not located in the heterochromatin [80] but, when ectopically targeted to heterochromatin, their ability of cluster heterochromatin compartments was rescued [81]. Interestingly, most MeCP2 mutations linked to disease showed impaired LLPS ability, including those that are directly involved in 5mC binding [58,59,74]. In addition, MeCP2 is able to recruit other important factors, such as ATRX required to stabilize the heterochromatin compartments and associated to regulation of genomic imprinting [82]. On the other hand, LLPS could be a mechanism to exclude competitor proteins. In the case of MeCP2-based LLPS, it could exclude proteins such as linker histone H1, albeit both are able to undergo LLPS [57]. In this context, it would be interesting to investigate whether and how heterochromatin factors recruit or exclude each other from the same compartments and how that regulates the organization of (hetero)chromatin in different tissues and cell types.

## Protein-histone dependent LLPS and function in heterochromatin

**Figure 6.**
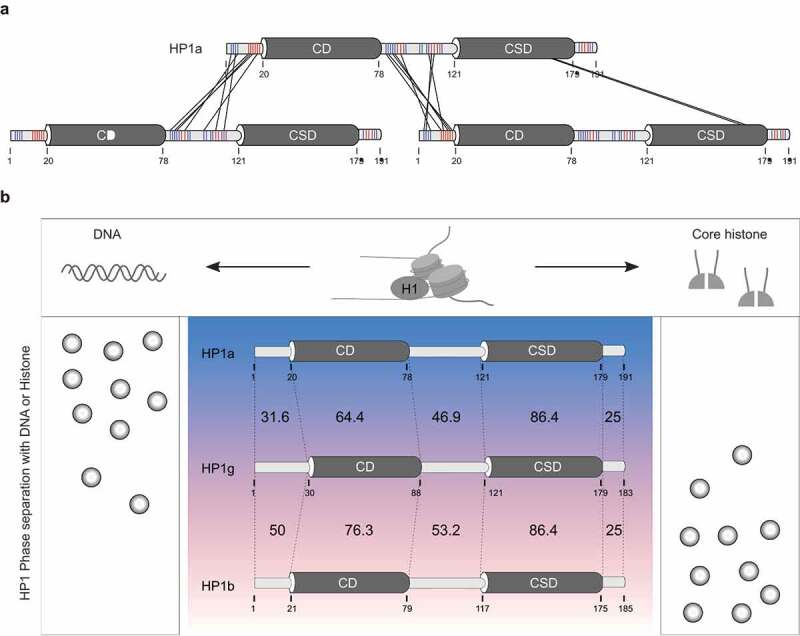
HP1-mediated heterochromatin phase separation.

Heterochromatin binding protein 1 (HP1) is a non-histone chromosome-binding protein [83]. HP1α, one of the three homologs of HP1 (termed HP1α, HP1β, and HP1γ) was the first heterochromatin-binding protein reported with the property of LLPS. This phase separation was shown to be initiated through intermolecular interaction of the phosphorylated HP1α N-terminus with the hinge region [13,84]. The dimerization mediated by the chromoshadow domain (CSD) also contributes to its LLPS (Figure 6a). HP1β and HP1γ are very conserved with HP1α in the chromodomain (CD) and CSD, and less conserved in the intrinsically disordered regions including the N-terminus, hinge region, and C-terminus (Figure 6b). The intrinsically disordered regions, especially the hinge region, determine the different isoelectric points of HP1 proteins that cause the different properties of HP1ʹs LLPS [64,85]. Both negatively charged DNA and positively charged histones bind HP1 proteins and affect their LLPS. Addition of DNA strongly promotes the LLPS of HP1α [85], while HP1β forms liquid-like droplets in the presence of core histones [64] (Figure 6b). However, HP1 itself was shown not to be required for heterochromatin compartmentalization [16,86], which is likely to be the case for every single heterochromatin-enriched protein. Notably, the linker histone H1 is excluded from the phase droplets of HP1β with core histones [64], but it can phase separate in the presence of DNA [57,87,88]. Addition of histone H1 promotes phase separation of polynucleosomes and slow dynamics of histones inside condensates [44]. HP1α-DNA, H1-DNA/polynucleosomes and HP1β-histones LLPS mechanisms contribute to heterochromatin formation and organization. The saturated histones or HP1 or DNA, lead to solid condensates [43,85,88]. Thus, LLPS of HP1 is highly regulated by its interacting partners including DNA and proteins. It was shown that the phosphorylation at the N-terminus of HP1α and the trimethylation of histone H3 lysine 9 (H3K9me3) are required for HP1α and HP1β LLPS, respectively [64,84]. The CSD domain mediates homo- and hetero-dimerization and is responsible for interaction with other proteins [89], while the CD domain of HP1 mediates recognition of H3K9me2 and H3K9me3 [90–92]. The interactome of HP1 range from heterochromatin structure regulators to cell cycle regulators, transcription, and DNA damage repair [93–95]. It was shown that the interacting proteins regulate the LLPS of HP1. For example, HP1 interaction with the inner nuclear membrane protein LBR (lamin B receptor) tethers heterochromatin to nuclear envelope [96]. This interaction negatively interferes with the multivalent interaction of HP1α and inhibits the LLPS of HP1α [84]. However, HP1 interaction with shugoshin 1 (Sgo1) targets it to centromeres in mitosis and protects centromeric sister-chromatid cohesion [97]. Addition of the peptides of Sgo1 responsible for this interaction promotes the LLPS of HP1α [84]. Thus, interacting proteins may regulate LLPS of HP1 and regulate chromatin dynamics during different cellular pathways. Importantly, HP1 phase separation correlates with the formation of heterochromatin and its clustering in the nucleus. HP1α and HP1β predominantly accumulate at pericentric heterochromatin (chromocenters), whereas HP1γ locates at euchromatin. Besides contributing to heterochromatin formation, HP1β was shown to trap its interactor KAP1 in the heterochromatin, thus regulating gene expression during loss of pluripotency [63]. This underscores the relevance of factor inclusion into LLPS compartments in addition to factor exclusion from LLPS compartments to the regulation of cell fate decisions.

(a) Phase separation of HP1α is driven by the multivalent interactions of the N-terminus and hinge region. The chromoshadow domain (CSD) domain mediated dimerization also contributes to HP1α phase separation. Color coding: blue for basic amino acids and red for acidic amino acids. CD, chromodomain. (b) Comparison of HP1 homologues in phase separation with DNA or histones. Protein sequence similarities from different regions are indicated. Colors from blue to pink code for the isoelectric point of proteins from high to low. H1 is excluded from the phase droplets of HP1β and core histones but can form LLPS with polynucleosomes by binding to DNA.

## Concluding remarks

As seen in the previous chapters, LLPS is one of the major driving forces for heterochromatin compartmentalization and *in vitro* experiments can be used to better understand how these membraneless compartments are formed *in vivo*. However, it is remarkable how small variations of any of the components of the reaction (proteins, salt, crowdedness) affect the final result of droplet formation or growth. Thus, it is increasingly important to establish standards in LLPS research and to quantitatively analyze the consequences caused by local perturbation (mutations, post-translational modifications, interactive network changes, etc.) both *in vitro* and *in vivo*. Only with such combined approaches, it will become possible to: i) compare results of different studies; ii) explore how LLPS dysfunctioning relates to (patho)physiology; and iii) elucidate how multiple factors come together or not to shape subcellular compartmentalization (e.g., of heterochromatin) and, as such, regulate cellular metabolism and cell fate decisions.
